# Locomotor adaptation to a powered ankle-foot orthosis depends on control method

**DOI:** 10.1186/1743-0003-4-48

**Published:** 2007-12-21

**Authors:** Stephen M Cain, Keith E Gordon, Daniel P Ferris

**Affiliations:** 1 Department of Biomedical Engineering, University of Michigan, 1107 Carl A. Gerstacker, 2200 Bonisteel Blvd., Ann Arbor, MI 48109-2099, USA; 2 Division of Kinesiology, University of Michigan, 401 Washtenaw Avenue, Ann Arbor, MI 48109-2214, USA; 3 Department of Physical Medicine and Rehabilitation, University of Michigan, Ann Arbor, MI 48109, USA; 4 Human Neuromechanics Laboratory, University of Michigan, 401 Washtenaw Avenue, Ann Arbor, MI 48109-2214, USA

## Abstract

**Background:**

We studied human locomotor adaptation to powered ankle-foot orthoses with the intent of identifying differences between two different orthosis control methods. The first orthosis control method used a footswitch to provide bang-bang control (a kinematic control) and the second orthosis control method used a proportional myoelectric signal from the soleus (a physiological control). Both controllers activated an artificial pneumatic muscle providing plantar flexion torque.

**Methods:**

Subjects walked on a treadmill for two thirty-minute sessions spaced three days apart under either footswitch control (n = 6) or myoelectric control (n = 6). We recorded lower limb electromyography (EMG), joint kinematics, and orthosis kinetics. We compared stance phase EMG amplitudes, correlation of joint angle patterns, and mechanical work performed by the powered orthosis between the two controllers over time.

**Results:**

During steady state at the end of the second session, subjects using proportional myoelectric control had much lower soleus and gastrocnemius activation than the subjects using footswitch control. The substantial decrease in triceps surae recruitment allowed the proportional myoelectric control subjects to walk with ankle kinematics close to normal and reduce negative work performed by the orthosis. The footswitch control subjects walked with substantially perturbed ankle kinematics and performed more negative work with the orthosis.

**Conclusion:**

These results provide evidence that the choice of orthosis control method can greatly alter how humans adapt to powered orthosis assistance during walking. Specifically, proportional myoelectric control results in larger reductions in muscle activation and gait kinematics more similar to normal compared to footswitch control.

## Introduction

Advancements in robotic technology have enabled several research groups around the world to build working robotic exoskeletons for assisting human locomotion [1-8]. The exoskeletons have a range of intended uses including enhancing human performance in healthy individuals, replacing motor capabilities in disabled individuals, and aiding in neurological rehabilitation. In each case, improvements in computer processing, energy efficiency, and sensors and actuators are allowing devices to far surpass previous expectations.

In order for robotic exoskeletons to better assist humans, it is imperative to determine how humans respond to mechanical assistance given by exoskeletons. Most of the published research has focused on hardware and software development. Few studies have actually measured human motor adaptation or physiological responses when using the devices. The human response is a key aspect that determines the success of the exoskeleton. Different exoskeleton control methods could produce extremely different levels of adaptation and adaptation rate, meaning that certain control schemes could prevent a user from effectively using an exoskeleton.

One of the main factors likely affecting how humans respond to mechanical assistance from an exoskeleton is the method of control. A wide range of control algorithms have been used by different research groups. They can rely on kinematic, kinetic, or myoelectric feedback, or some combination of these [3,7-15]. Because each research group has their own custom-built hardware along with their own control algorithm, it would be difficult to separate the effects of controller from hardware even if human response results were readily available in the literature.

We developed a single-joint ankle exoskeleton (i.e. powered ankle-foot orthosis) that can supply mechanical plantar flexion assistance during walking [14-17]. For this study, we studied locomotor adaptation in healthy subjects walking with the powered ankle-foot orthosis using two different orthosis control methods. By using the same exoskeleton to evaluate each orthosis control method, we can separate the effects of the controller from the hardware. One group of subjects used footswitch control that activated the orthosis when the forefoot made contact with the ground [16]. A second group of subjects used proportional myoelectric control that activated the orthosis based on soleus electromyography amplitude [14,18]. The two orthosis control methods were chosen based on our previous experience and familiarity with how they could be used with our specific exoskeleton. The footswitch control is a simple and purely kinematic/kinetic orthosis control method, depending only upon the gait kinematics of the subject and the forces acting on the foot during gait. The proportional myoelectric control is an orthosis control method depending only upon the subject's motor commands.

The purpose of this study was to directly compare human responses to a robotic exoskeleton using two different orthosis control methods. The two control methods affect the relationship of the efferent signal to movement in different ways. In footswitch control the supplied exoskeleton torque and the efferent signal are not well related – existence of muscle activation or motor commands does not guarantee that the exoskeleton is producing torque. In proportional myoelectric control, the supplied exoskeleton torque is related directly to the motor command. We hypothesized that different control methods (footswitch versus proportional myoelectric) used to control a powered ankle-foot orthosis would produce differences in how subjects adjusted gait kinematics and muscle activation to adapt to the powered exoskeleton.

## Methods

Twelve healthy subjects [(mean ± standard deviation) 6 male, 6 female, age 25.15 ± 2.5 years, body mass 74.1 ± 11.84 kg] gave informed consent and participated in the study. The University of Michigan Medical School Institutional Review Board approved the protocol.

### Hardware

**Figure 1:**
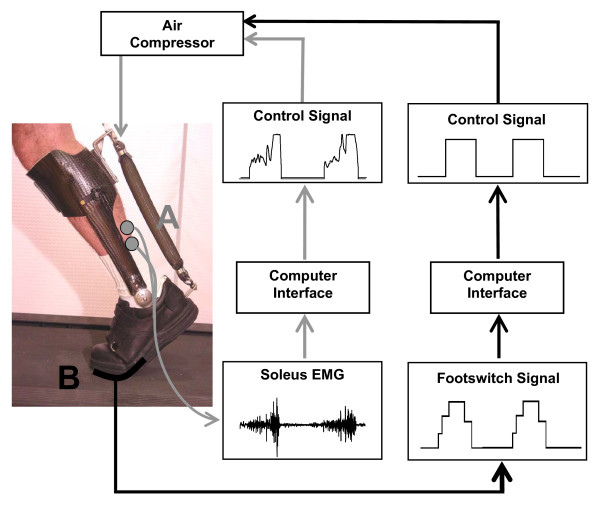
**Two orthosis control methods**. Two control schemes (A, gray arrows: proportional myoelectric control, and B, black arrows: footswitch control) were used to activate the artificial pneumatic muscle. This pneumatic muscle was fastened to the shank and heel sections of a carbon fiber ankle-foot orthosis that allowed free sagittal plane rotation at the ankle joint. When activated, this muscle produced a plantar flexion torque at the ankle.

We fabricated a custom ankle-foot orthosis (AFO) for each subject's left leg (Figure 1). Construction and testing of the AFO has been described in detail [14-16]. Each AFO consisted of a carbon fiber shank section and polypropylene foot section. A metal hinge joining the shank and foot sections permitted free sagittal plane rotation of the ankle. Each orthosis weighed approximately 1.1 kilograms, which adds distal mass to a subject's left leg. The added distal mass likely slightly increased the metabolic cost of walking [19]. The passive orthosis also slightly affected subjects' ankle kinematics, causing slightly increased plantar flexion (<1 degree) during swing.

We attached a pneumatic artificial muscle to the posterior of each AFO. Inflating (pressurizing) the pneumatic muscle created a plantar flexor torque. The artificial pneumatic plantar flexor muscle had a moment arm of approximately 10 centimeters. Air was supplied to the pneumatic muscle by four parallel proportional pressure regulators (MAC Valves, Inc., Wixom, MI) via nylon tubing (0–6.2 bar). An analog-controlled solenoid valve (MAC Valves, Inc., Wixom, MI) was attached in parallel with the air supply to assist in exhausting unwanted air from the pneumatic muscle. Pressurization of the pneumatic muscle and solenoid valve activity produced sounds that were audible to the subject.

### Testing protocol

Subjects completed two identical sessions of testing wearing the AFO. Each session went as follows: 10 minutes of treadmill walking with the AFO passive (**Passive AFO**), 30 minutes of treadmill walking with the AFO powered (**Active AFO**), and finally 15 minutes of walking with the AFO passive (**Passive AFO**). The transitions from passive to powered, and powered to passive, occurred without stopping. For safety, we gave the subject an oral countdown to when the transition would occur. The second session of testing was completed three days after the first session. This three day rest period was chosen to allow the subjects to recover from any muscle fatigue and soreness that may have occurred during the first session.

All subjects were naive, never experiencing walking with a powered orthosis until the first day of training. Before testing, subjects were told that the powered orthosis would provide "extra push-off force." We instructed subjects to walk in the manner they preferred and that it would take some time to adjust to the powered orthosis.

### Control

The pressure in the pneumatic muscle was controlled by one of two real-time control schemes: proportional myoelectric control or foot switch control (Figure 1). Subjects experienced either proportional myoelectric control or foot switch control (six subjects, 3 male and 3 female, in each control scheme).

In the footswitch control scheme, we controlled the pressure in the pneumatic muscle through the use of a forefoot footswitch (B & L Engineering, Tustin, CA). This footswitch control was implemented through a desktop computer and a real-time control board (dSPACE, Inc., Northville, MI). The software was composed in Simulink (The Mathworks, Inc., Natick, MA) and converted to ControlDesk (dSPACE, Inc., Northville, MI). The software sent a 0 to 10 V analog signal to the proportional pressure regulators and solenoid valves to control the activation and deactivation (pressure) of the pneumatic muscles. The software program regulated air pressure in the pneumatic muscle via an on-off or "bang-bang" controller. If the voltage signal from the footswitch was below the threshold value (a threshold was used to ensure a consistent pressure control signal), then the software signaled for zero or minimum pressure in the pneumatic muscle. If the voltage signal was above the threshold, the software signaled for maximum pressure in the pneumatic muscle.

In the proportional myoelectric control scheme, the pressure in the pneumatic muscle was proportional to the processed soleus electromyography (EMG). The EMG signal was processed as follows: It was first high-pass filtered with a second-order Butterworth filter (cutoff frequency 20 Hz) to remove movement artifact, full wave rectified, and low-pass filtered with a second-order Butterworth filter (cutoff frequency 10 Hz) in order to smooth the signal. Setting threshold cutoff values appropriately eliminated background noise in the signal. The amplitude of the control signal was scaled with adjustable gains. The control was implemented in the same way as the footswitch control except that the control signal was proportional. Data from the six subjects who used proportional myoelectric control was previously reported by Gordon and Ferris [18].

Because the control signal that resulted from the myoelectric control scheme was proportional, it was important to set the gain of the control signal consistently. We tuned the gain separately each day to ensure that the relationship between the soleus EMG and the control signal remained the same. To set the gain, we followed the following procedure: 1) While the subject walked with the AFO passive (the first Passive AFO period), we adjusted the gain without activating the AFO so that a maximum control signal (10 V) was produced at the maximum or peak of the soleus EMG. 2) We then doubled the gain. 3) After doubling the gain, we did not change it for the remainder of the training session.

It is important to note that there is not a simple linear relationship between the control signal amplitude (whether it is from electromyography or a footswitch) and the force developed by the muscle/torque provided by the orthosis. The control signal directly controlled the pressure supplied to the pneumatic muscle. Increasing pressure in the muscle increases the force developed by the muscle. However the force that the muscle actually develops is affected by its activation (pressure), the muscle length, and the bandwidth [16]. In isometric conditions, a pneumatic muscle is able to develop 1700 N of force. As the muscle shortens, less force is developed. When the muscle reaches its minimum length (~71% of its resting length), the force developed drops to zero. The force bandwidth of the artificial muscle is approximately 2.4 Hz, which is very similar to the 2.2 Hz force bandwidth of human muscle [20]. Approximately a 50 ms electromechanical delay existed between onset of the control signal and the initial rise in the artificial muscle tension. A more detailed description of the pneumatic muscle performance can be found in Gordon *et al.*[16]. There is no direct relationship between the control signal and the force/torque provided by the AFO. Therefore, a bang-bang control signal does not result in an applied bang-bang torque or power at the ankle joint.

### Data collection

We recorded kinematic, kinetic, and electromyography data from each subject during the first 10 seconds of every minute as they walked on a treadmill at 1.25 m/s. Kinematic data was sampled at 120 Hz. All other signals were sampled at 1200 Hz. Three-dimensional kinematic data was recorded using a 6-camera video system (Motion Analysis Corporation, Santa Rosa, CA) and twenty-nine reflective markers placed on each subject's pelvis and lower limbs. Step cycle data was collected using footswitches (B & L Engineering, Tustin, CA), which were placed in each shoe. Artificial pneumatic muscle force was measured using a compression load cell (Omega Engineering, Stamford, CT) mounted in series with the pneumatic muscle. We recorded lower limb surface EMG (Konigsberg Instruments, Inc., Pasadena, CA) from the left soleus, tibialis anterior, medial gastrocnemius, lateral gastrocnemius, vastus lateralis, vastus medialis, rectus femoris, medial hamstring and lateral hamstring muscles using bipolar surface electrodes. The EMG was bandpass filtered with a lower bound of 12.5 Hz and an upper bound of 920 Hz. We minimized crosstalk by visually inspecting the EMG signals during manual muscle tests prior to treadmill walking, moving electrode placement if needed. We marked the position of the electrodes on each subject's skin using a permanent marker to ensure the same electrode placement for the second session of testing. The sound of the pneumatic muscle inflating and deflating was audible to the subjects for both control signals. No distinguishable difference between the noises associated with each controller could be identified.

### Data analysis

We created average step cycle profiles of each minute of walking for EMG, kinematic, and kinetic variables for each subject. Each minute's average step cycle was calculated from the complete step cycles that occurred during the first 10 seconds of that minute. To examine how EMG amplitude changed over time, we calculated the normalized root mean squared (RMS) EMG values for each minute of walking for each subject. RMS EMG values were calculated from high pass filtered (cutoff frequency 40 Hz) and rectified EMG data for the complete gait cycle, stance phase, and swing phase. All RMS EMG values were normalized to the last minute of walking with the passive AFO before activating the pneumatic muscle (the last pre-passive minute), or what we called the **Baseline **condition. We also made average step profiles for the joint angles that were created from the marker data (low-pass filtered, cutoff frequency 6 Hz). In order to examine the changes in the kinematics over time, we calculated joint angle correlations between the average step cycle profiles of each minute and the average joint profile from the last pre-passive minute for the same session. We created average step cycle torque and power profiles for the AFO only (torque and power that the AFO was producing). From these, we calculated the positive and negative work performed by the AFO during a step cycle. Foot and shank parameters were adjusted to account for added AFO mass and inertia.

**Figure 2:**
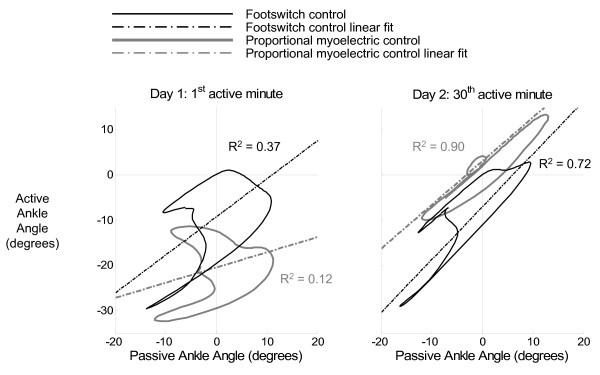
**Ankle angle correlation common variance**. The plots above compare the two controllers (footswitch control = black, proportional myoelectrical control = gray) and their effect on ankle kinematics during the subjects' first experience with the powered orthosis (day 1, 1^st ^active minute) and the end of training (day 2, 30^th ^active minute) for all 12 subjects (n = 6 for each control scheme). On the first day during the first minute, the ankle kinematics changed significantly regardless of the controller used. Initially, the proportional myoelectric control resulted in more perturbation at the ankle than the footswitch control. At the end of training, subjects returned closer to normal (baseline) kinematics regardless of controller. Proportional myoelectric control resulted in more normal kinematics than footswitch control.

Four parameters were used to assess the adaptation rate and degree of adaptation: soleus EMG RMS during the stance phase, ankle angle correlation common variance, positive orthosis work, and negative orthosis work. Soleus EMG RMS during stance was chosen to assess how the neural control of the subjects changed over the training period. Ankle angle correlation common variance was selected to measure how the kinematics of the walking pattern changed (Figure 2). Ankle angle correlation common variance was calculated for each minute by plotting the ankle angle of that minute versus the ankle angle during the last minute of passive walking before activating the orthosis (the Baseline condition). A linear fit of active versus passive ankle angle was calculated for each minute, and a R^2 ^correlation value was found for each linear fit. Positive and negative work allowed us to evaluate how effectively subjects were able to use the powered orthosis.

### Statistics

We used a general linear model (GLM), or multiple regression, to test for significant effects between controllers, effects of minute within footswitch control group, and effects of minute within proportional myoelectric control group for the four outcome parameters (soleus EMG RMS, ankle angle correlation common variance, positive orthosis work, and negative orthosis work). The equation for the general linear model is of the form y = β_0 _+ β_1_x_1 _+ β_2_x_2 _+ ... + β_n_x_n _+ ε, where Y is the response variable, β_n _are model parameters, and ε is the error. Our previous study examining subjects using proportional myoelectric control found that subjects were at steady state walking dynamics for the last 15 minutes of powered orthosis walking on the second day of training [18]. As a result, we used only the last 15 minutes of data on day 2 to test for significant differences between controllers during steady state. A general linear model was also used to test the effect of controller on post-adaptation, or the period of walking after turning the power to the AFO off. The entire 15 minutes of post-powered orthosis walking was used for the post-adaptation analysis.

To test for differences in adaptation rate between controllers, we used the methodology of Noble and Prentice [21]. This method defines a band of normal variation within steady state dynamics and then calculates the amount of time required to reach and stay within that band. As mentioned above, we used data from the last 15 minutes of powered walking on day two for the steady state period. The band of steady state variation for each outcome parameter was calculated as the mean ± two standard deviations from the steady state period. Time to steady state was defined as the time it took for a measure to enter the steady state range and remain there for three consecutive minutes without any two consecutive minutes outside of the steady state range afterwards. This analysis was performed for each subject individually. Differences in learning rate (time to steady state) were assessed using a repeated measures ANOVA.

### Overground walking

An overground testing session was used to measure the amount of work and power that each subject produced without the AFO. This let us estimate the amount of assistance that the powered AFO was providing the subjects. During the overground collection, a subject would walk without wearing an AFO over two force plates at a speed of 1.25 m/s (± 0.06 m/s). Subjects completed ten trials. Force plate data and kinematic marker data were used to calculate net torques and work performed about the ankle joint by using commercial software (Visual3D, C-Motion, Inc., Rockville, MD).

## Results

### Effects and responses

**Figure 3:**
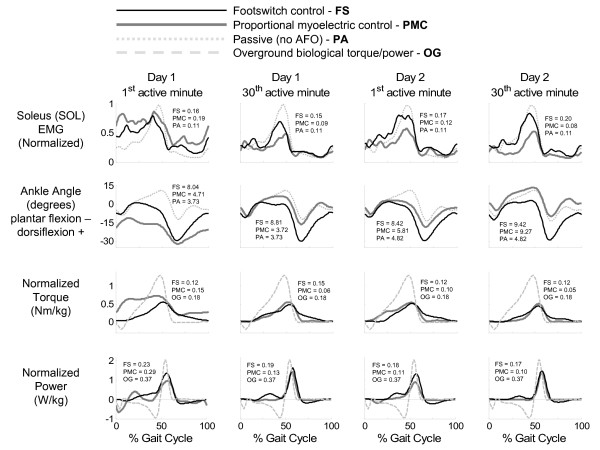
**Effects of the powered ankle-foot orthosis on soleus muscle activation, sagittal ankle angle, orthosis torque, and orthosis power under each control scheme**. The effects of the powered ankle-foot orthosis on soleus muscle activation, sagittal ankle angle, orthosis torque, and orthosis power under each control scheme (footswitch control = thin black line, proportional myoelectrical control = thick gray line) are shown for the first and last minutes of powered walking for both days. Soleus muscle activation and ankle angle are plotted with passive (normal) data (light gray dotted line) for comparison. Orthosis torque and power are plotted with normal overground biological torque and power (light gray dashed line). Electromyography is normalized to the peak Baseline (passive) value. After two training sessions, subjects using footswitch control continued to walk with increased plantar flexion whereas subjects using proportional myoelectric control reached more normal ankle kinematics (as measured by ankle angle correlation common variance). The powered ankle-foot orthosis was able to supply approximately forty percent of the biological ankle torque. Data shown is from all 12 subjects (n = 6 for footswitch control, n = 6 for proportional myoelectric control, n = 12 for passive data). The average standard deviation over the stride cycle for each signal and each condition is reported in each plot in units consistent with that signal.

**Figure 4:**
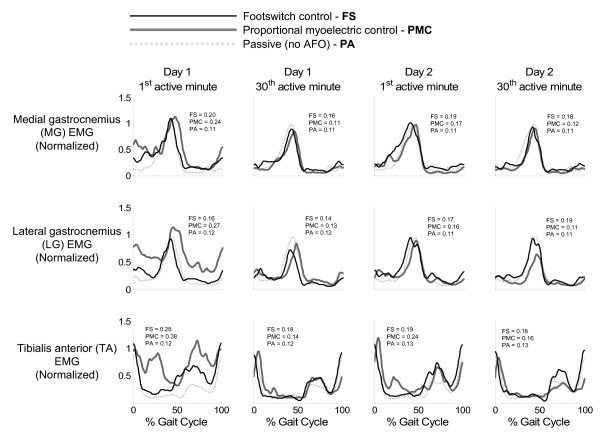
**Effects of the powered ankle-foot orthosis on lower leg muscles**. Average medial gastrocnemius (MG), lateral gastrocnemius (LG), and tibialis anterior (TA) muscle activations are plotted alongside passive orthosis muscle activations for the first and last minutes of powered orthosis walking for both days of training and both controllers [footswitch control (FS) = thin black line, and proportional myoelectric control (PMC) = thick gray line]. Electromyographies are normalized to the peak passive values. By the end of the second day of training, muscle activation patterns were not much different from normal (light gray dotted line). Each plot is the average of multiple subject data: 6 subjects for all footswitch control data, 5 subjects for proportional myoelectrical control MG and LG, 4 subjects for proportional myoelectrical control TA. The average standard deviation over the stride cycle for each signal and each condition is reported in each plot in units consistent with that signal.

**Figure 5:**
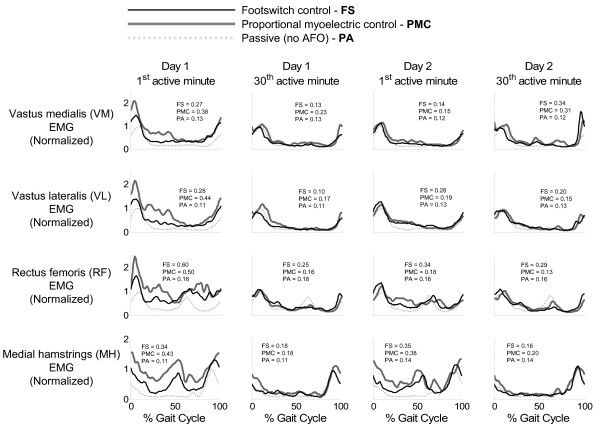
**Effects of the powered ankle-foot orthosis on upper leg muscles**. The vastus medialis (VM), vastus lateralis (VL), rectus femoris (RF), and medial hamstrings (MH) muscle activations are plotted alongside passive orthosis muscle activations for the first and last minutes of powered orthosis walking for both days of training and both controllers [footswitch control (FS) = thin black line, and proportional myoelectric control (PMC) = thick gray line]. Electromyographies are normalized to the peak passive values. By the end of the second day of training, muscle activation patterns returned very close to normal (light gray dotted line). Each plot is the average of multiple subject data: 6 subjects for all footswitch control data, 6 subjects for proportional myoelectrical control MH, 5 subjects for proportional myoelectrical control VL and RF, 4 subjects for proportional myoelectrical control VM. The average standard deviation over the stride cycle for each signal and each condition is reported in each plot in units consistent with that signal.

The walking patterns of the subjects changed substantially when the AFO provided additional plantar flexion torque at the beginning of training. The initial changes were substantial regardless of the controller used. When first experiencing the powered AFO condition (minute 1, day 1), the extra torque caused the subjects to walk with increased plantar flexion. This plantar flexion was greatest at toe-off, where it was approximately 17 degrees greater than unpowered orthosis walking. The significant initial change in ankle kinematics was also reflected in the ankle angle correlation common variance, which decreased from 1 during unpowered walking to 0.37 and 0.12 for footswitch orthosis control and soleus proportional myoelectrical orthosis control, respectively (Figure 2). Subjects also initially demonstrated increased muscle activation throughout the stance phase (Figures 3, 4, 5).

Muscle activation patterns were modified as the subjects trained with the powered AFO. Examples of these changes can be seen in Figures 4 and 5. By the end of the second day of training, differences in the muscle activation patterns compared to passive orthosis walking were very subtle. The exception to this was the soleus muscle activation amplitude in the subjects using proportional myoelectric control (Figure 3). There were no significant differences in stride time between orthosis control methods, condition, or day. Footswitch subjects had a stride time of 1.26 ± 0.10 seconds (mean ± standard deviation) and proportional myoelectric subjects had a stride time of 1.24 ± 0.12 seconds. The artificial plantar flexor produced a peak torque that was approximately 47% of the peak torque generated at the ankle when walking overground (Figure 3). As subjects trained with the powered AFOs, the torque and power produced by the AFO became more focused at toe-off (Figure 3).

### Learning rates

**Figure 6:**
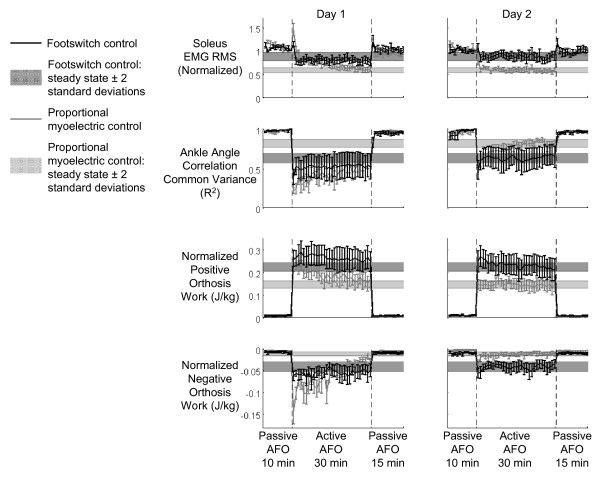
**Soleus EMG RMS, ankle angle correlation common variance, positive orthosis work, and negative orthosis work changes across both training sessions**. Soleus EMG RMS, ankle angle correlation common variance, positive orthosis work, and negative orthosis work are plotted (mean ± standard error) across both training sessions for each minute. Results for each controller [footswitch control = black line and dark shading, proportional myoelectrical control = gray line and light shading] are shown along with the steady state band for each measure. Time till steady state was used as a measure of the adaptation rate. Differences in day 1 versus day 2 adaptation rates were significant (ANOVA, p < 0.005). On day 2, footswitch control resulted in faster adaptation in negative orthosis work (GLM, p = 0.0115). At steady state, proportional myoelectric control resulted in less soleus activation (GLM, p = 0.0342), closer to normal ankle kinematics (GLM, p = 0.0417), and less negative work (GLM, p = 0.0085) than footswitch control. The steady state envelopes displayed are calculated for the group mean data (n = 6 for each controller) for display purposes only; individual subject analyses were calculated in the same way and were used for statistical tests.

**Figure 7:**
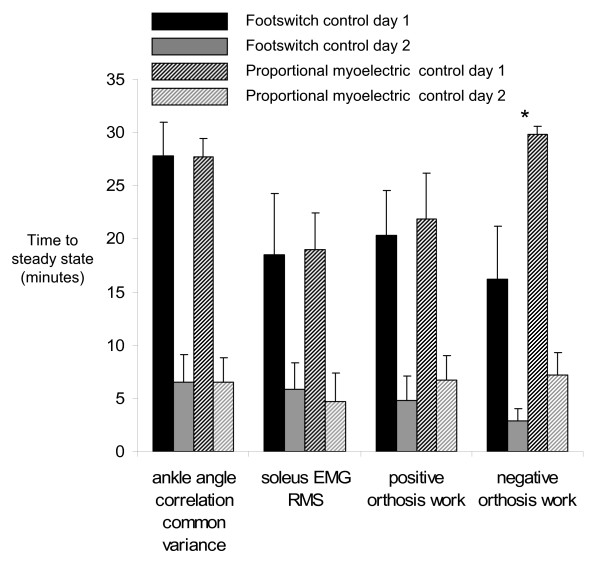
**Adaptation rates**. Adaptation rates expressed as time in minutes to steady state for ankle angle correlation common variance, soleus EMG RMS, positive orthosis work, and negative orthosis work are plotted for each controller and each day (mean + standard error) [footswitch control = solid bars, proportional myoelectrical control = hashed bars]. Significant differences in time to steady state were found between days for each controller (GLM, p < 0.005). The only difference in between controllers was the adaptation rate of the negative orthosis work, in which the footswitch control was faster (GLM, p = 0.0115). (asterisk) indicates significant difference between studies.

There were significant differences in learning rates between days, but few significant differences in learning rates between controllers. All four of the movement parameters (soleus EMG RMS, ankle angle correlation common variance common variance, positive orthosis work, negative orthosis work) showed significant differences by day (ANOVA, p < 0.005). For each measure and both controllers, steady state was reached more quickly on the second day of training (Figures 6 and 7). The only significant difference in learning rate between controllers was in negative orthosis work. Subjects reached negative orthosis work steady state more quickly when using footswitch control than when using proportional myoelectric control (ANOVA, p = 0.0115).

### Steady state

**Figure 8:**
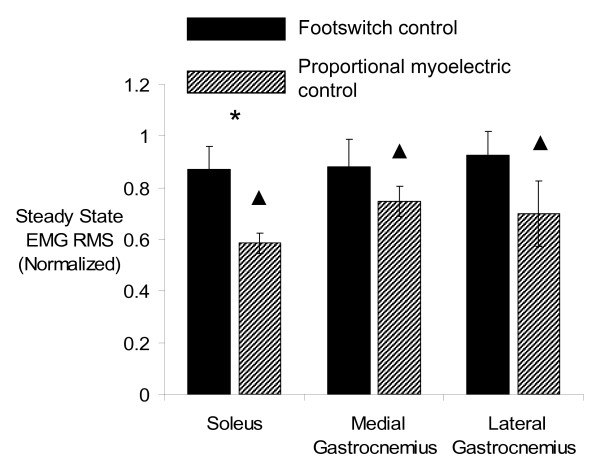
**Steady state muscle activation**. Steady state EMG RMS values of the soleus, medial gastrocnemius, and lateral gastrocnemius are plotted for each controller and each day (mean ± standard error) [footswitch control = solid bars, proportional myoelectrical control = hashed bars]. Average data is used for each plot (n = 6), except for the proportional myoelectric control medial gastrocnemius (n = 5) and lateral gastrocnemius (n = 5). The RMS values are normalized by dividing by the RMS of the passive orthosis condition. Proportional myoelectric control resulted in less muscle activation for the soleus than footswitch control (GLM, p = 0.0144). (asterisk) indicates significant difference between studies. (triangle) indicates significant difference from baseline (GLM, p < 0.03).

The last 15 minutes of powered orthosis walking were found to be constant (no change in movement parameters with time) for both controllers and all movement parameters except ankle angle correlation common variance and negative orthosis work when using footswitch control. Time was found to have a significant effect on both measurements (ankle angle correlation common variance p = 0.0417, negative orthosis work p = 0.0085), however the rates of change were very small (ankle angle correlation common variance slope = 0.0058 units/min, negative orthosis work slope = 0.00051 J/kg/min). Differences in the steady state walking patterns were found between controllers. Subjects using proportional myoelectric control reduced steady state EMG amplitudes of the soleus more than subjects who used footswitch control (GLM, p = 0.0144, Figure 8). Subjects using proportional myoelectric control walked with ankle kinematics (as measured by ankle angle correlation common variance) closer to baseline than subjects using footswitch control (GLM, p = 0.0417). At steady state, more negative orthosis work was produced by subjects using footswitch control (GLM, p = 0.0085). There was a trend for subjects using footswitch control to also produce more positive orthosis work but it was not statistically significant (GLM, p = 0.0575).

Subjects using both controllers walked with kinematics different from baseline (GLM, p < 0.03). Only subjects using proportional myoelectric control reduced EMG amplitudes of the soleus, medial gastrocnemius, and lateral gastrocnemius below baseline (GLM, p < 0.03). It is important to note that Gordon and Ferris [18] only found that the soleus EMG amplitude was significantly different from baseline for subjects (n = 10) using proportional myoelectric control.

### Post-passive adaptation

No significant differences in post-passive adaptation rate were found between the two controllers.

## Discussion

Subjects using proportional myoelectric control returned closer to their normal (Baseline) kinematic patterns by the end of the second day compared to subjects using footswitch control. There are several aspects of the proportional myoelectric control that could have contributed to this difference. First, proportional control allows for a more graded response in orthosis dynamics than the bang-bang nature of footswitch control used in this study. With step-to-step variability in orthosis output, it would likely be easier for the nervous system to determine the relationship between soleus activation and orthosis assistance using proportional myoelectric control than using footswitch control. Second, proportional myoelectric control put the orthosis under a control mode that is more similar to the normal physiologic control that the nervous system uses to generate motion. It is likely that the nervous system has some representation of the transfer function from soleus motor neuron recruitment to ankle movement. Wearing the orthosis with proportional myoelectric control would likely be interpreted as a relatively minor change in the transfer function. Wearing the orthosis with footswitch control would likely be a more non-natural modification to lower limb movement control.

**Figure 9:**
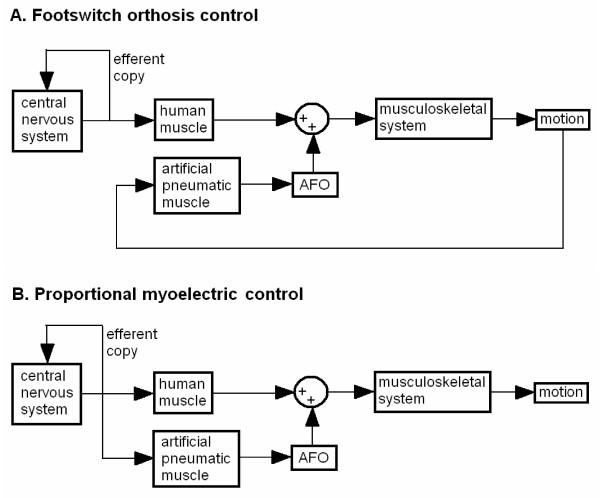
**Comparison of control methods**. Comparing simplified block diagrams of footswitch (A) versus proportional myoelectric (B) control reveals a fundamental difference in the neurological integration of the control signal. In each control method, physiological muscle and the artificial pneumatic muscle act on the musculoskeletal system to create motion. A: In footswitch control the control signal for the artificial pneumatic muscle is generated by a footswitch. The signal from the footswitch depends on the motion (kinematics) of the subject. The efferent copy cannot provide the central nervous system with precise information about when the artificial pneumatic muscle is active or how strong it is contracting. B: In proportional myoelectric control the control signal for the artificial pneumatic muscle is generated by the subject's electromyographic activity. This signal is closely related to the efferent signal sent to physiological muscle from the central nervous system. The efferent copy therefore provides the central nervous system with information about what the control signal is and can be used effectively by the central nervous system to make predictions about what the artificial pneumatic muscle is doing.

Both of the possibilities are dependant upon the relationship between the efferent and afferent signals to the movement generated by the orthosis. With both controllers, the sensory signals or afferent signals are used by the central nervous system to estimate the system's state. However, the efferent signals or motor control signals must also be used to make predictions about the system to control movement [22]. With proportional myoelectric control, the motor control signal is closely related to the orthosis behavior, allowing for accurate prediction (Figure 9b). With footswitch control, the orthosis control signal is not related well to any motor control signals (Figure 9a). The footswitch control has different effects, depending on whether the foot is on the ground or in the air. This could be thought of as trying to learn two different dynamics at once – each is presented in rapid succession. Rapid succession of two dynamic systems interferes with motor learning [22]. We cannot separate out the relative importance of the two possibilities with the data from this study, but it is clear that the choice of controller can have substantial effects on the walking pattern.

The artificial pneumatic plantar flexor produced a peak torque 47% of the maximum ankle plantar flexor torque produced when walking (Figure 3). We did not expect the powered orthosis to provide all of the torque needed at the ankle during gait. In a previous study by Gordon *et al.*[16] the powered orthosis was only able to generate a peak plantar flexor torque that was 57% of the peak net ankle plantar flexor moment, regardless of the potential force generation capabilities of the artificial pneumatic plantar flexor. Gordon *et al.*[16] also found that the net ankle moment remained approximately the same regardless of the assistance given to the subjects; the sum of the AFO produced torque plus the physiological torque was approximately equal to the physiological torque produced when walking without a powered orthosis. A good estimate of what torque the ankle is producing is the difference between overground biological torque and the torque produced by the powered orthosis (Figure 3). Previously, the powered orthosis was found to produce about 70% of the positive plantar flexor work done during normal walking [16].

It is possible that the footswitch control signal was producing too much torque (more than required for normal walking). Reducing the magnitude of the bang-bang control signal used for the footswitch control method could allow a new dynamic equilibrium point closer with normal or baseline kinematics and reduced plantar flexion activation.

The differences in soleus activation between the two controllers (Figure 8) suggest that proportional myoelectric control may lead to a lower metabolic cost of transport than the footswitch control. Muscle activation requires the use of metabolic energy. Although nonlinear factors such as muscle length and velocity will affect the relationship between muscle recruitment and metabolic cost [23], the larger reductions in plantar flexor muscle recruitment for proportional myoelectric control compared to footswitch control may override the differences in muscle-tendon kinematics. This is an important possibility to consider given recent findings from Norris *et al.*[24]. They showed that the metabolic cost of transport decreased by about 13% when subjects walked with two powered AFOs similar to the design used in this study [24]. However, Norris *et al.*[24] used a bang-bang control algorithm that started and stopped orthosis activation based on the angular velocity of the foot. Thus, this type of control was similar to our footswitch control; it depended on motion and not neurological signals. It seems feasible that proportional myoelectric control might reduce the metabolic cost of transport during walking more than 13%.

The two controllers produced similar adaptation rates for most parameters. The only significant difference in adaptation rates between controllers was for negative orthosis work. Subjects using footswitch control reached steady state faster on both days of training compared to subjects using proportional myoelectric control. Regardless of control mode, subjects adapted to the powered orthosis much more quickly on the second day. This indicates that subjects were able to store a motor memory of how to walk with the orthosis and then recall that motor memory on a later date. The controller used did not seem to affect this formation or recall of the motor memory.

The results from this study may have been altered if subjects had been allowed to practice using the orthosis for a longer time period. Additional days of training might have resulted in further adaptation to the walking pattern. However, given the relative steady state nature of the outcome parameters during the last 15 minutes of day two (Figure 6), any additional changes would have likely required multiple days.

## Conclusion

The choice of controller for a robotic exoskeleton can have a substantial effect on human-machine performance. If the goal of the exoskeleton is to walk with relatively normal joint kinematics, proportional myoelectric control should be preferred over footswitch control. In addition, it seems reasonable to suggest that proportional myoelectric control may provide metabolic savings greater than those from footswitch control as well.

The findings of this study also have important implications for rehabilitation. While rate of motor adaptation was not affected by controller, the steady state walking dynamics were more similar for proportional myoelectric control than footswitch control. This suggests that robotic devices designed to facilitate adaptive training may benefit from more direct nervous system control. Proportional myoelectric control may also have the benefit of amplifying movement errors during practice. Patton *et al.*[25] found that practice with error-enhancing mechanical forces was more effective in improving movement ability of stroke subjects compared to practice with error-reducing mechanical forces. It would be very interesting to examine how patients with neurological deficits responded to walking practice with a powered orthosis under proportional myoelectric control. It could improve motor learning by enhancing errors in neuromuscular activation patterns in a manner to that found by Patton *et al.*[25]. Future studies are needed to examine this possibility.

## Competing interests

The author(s) declare that they have no competing interests.

## Authors' contributions

SMC recruited subjects, managed data collections, and completed data analysis for the footswitch orthosis control method. KEG recruited subjects, managed data collections, and completed data analysis for the proportional myoelectric orthosis control method. SMC completed all data analysis for comparing the two orthosis control methods, and drafted the manuscript. KEG edited the manuscript. DPF conceived the study, provided guidance on experimental design, assisted with data collections, and edited the manuscript. All authors read and approved the final manuscript.
